# Whole-Genome Sequence of a Reemerging Listeria monocytogenes Serovar 1/2a Strain in Central Italy

**DOI:** 10.1128/MRA.01069-18

**Published:** 2018-12-13

**Authors:** Massimiliano Orsini, Marina Torresi, Claudio Patavino, Patrizia Centorame, Antonio Rinaldi, Vicdalia Aniela Acciari, Gabriella Centorotola, Maurilia Marcacci, Massimo Ancora, Marco Di Domenico, Giuliana Blasi, Anna Duranti, Samuel Perticara, Adriano Di Pasquale, Cesare Cammà, Francesco Pomilio, Giacomo Migliorati

**Affiliations:** a Istituto Zooprofilattico Sperimentale dell’Abruzzo e del Molise, National Reference Laboratory for *Listeria monocytogenes*, Teramo, Italy; b Istituto Zooprofilattico Sperimentale dell’Umbria e delle Marche, Perugia, Italy; Queens College

## Abstract

We report the whole-genome sequence of a Listeria monocytogenes strain isolated from a child in central Italy. Interestingly, the sequence showed a difference of only 13 single-nucleotide polymorphisms (SNPs) from a strain responsible for a severe listeriosis outbreak that occurred between January 2015 and March 2016 in the same region.

## ANNOUNCEMENT

Listeria monocytogenes is a foodborne bacterial pathogen (1). In the period from January 2015 to March 2016, a severe invasive listeriosis outbreak that caused several deaths occurred in central Italy (2). The strain responsible for the outbreak exhibited a peculiar pulsed-field gel electrophoresis (PFGE) pattern (AscI.0183 ApaI.0063) and was recently published (3). Recently, in March 2018 in the same region, a new case of listeriosis affecting a child (female, 10 months old) who developed septicemia was reported. The strain was isolated from peripheral blood according to the ISO 11290 procedure (reviewed in reference [4]). The strain was then plated on blood agar and incubated at 37°C for 24 h for nucleic acid extraction. DNA extraction was performed with the Maxwell 16 tissue DNA purification kit (Promega Italia Srl, Milan, Italy) according to the manufacturer's protocol. Characterization results highlighted a Listeria monocytogenes serovar 1/2a strain showing the same PFGE pattern as that found in the previous outbreak. We used 1 ng of genomic DNA for library preparation with the Nextera XT DNA library prep kit (Illumina, San Diego, CA) according to the manufacturer’s protocol. Deep sequencing was performed on the NextSeq 500 platform (Illumina) with the NextSeq 500/550 mid output reagent cartridge v2 (300 cycles, standard 150-bp paired-end reads).

The complete genome of strain 2018TE5305-1-4 was obtained on the NextSeq 500 platform, and we obtained 8,821,656 reads (average length, 150 nucleotides), corresponding to a theoretical coverage of about 450×. Quality control was performed with FastQC (http://www.bioinformatics.babraham.ac.uk/projects/fastqc/), and trimming was carried out with a command line version of the trimming tool (FastQ positional and quality trimming) available on the Orione Web server (5). Genome assembling was performed by mapping with the 2015TE24968 strain genome sequences (GenBank accession number NZ_CP028406) as a template and Bowtie 2 (6) as an aligner, followed by the *samtools-bcftools-vcfutils* pipeline (https://github.com/samtools). The genome sequence was refined by remapping at high stringency the trimmed reads against the obtained draft using the ungapped aligner Bowtie (7). The alignment file was converted in *vcf* format, and any returned variant satisfying minimum criteria (coverage higher than 8, mapping quality higher than 30, and no alternative variants) was incorporated into the draft sequence. The whole-genome sequence, which assembled in a single circular contig (length, 2,894,716 bp), was submitted to GenBank and annotated using the PGAP (https://www.ncbi.nlm.nih.gov/genomes/static/Pipeline.html). Annotation returned 2,949 genes, 2,829 coding sequences, 6 full rRNA operons, 31 pseudogenes (21 of them were frameshifted genes), 2 CRISPR arrays, 4 noncoding RNAs (ncRNAs), and 67 tRNAs. A full-length plasmid (length, 57,530 bp) coding for 62 genes and 2 pseudogenes also was found, which was identical to the plasmid pl2015TE24968 isolated in 2015 (GenBank accession number NZ_CP015985).

Whole-genome alignment with the strain isolated in 2015 (GenBank accession number NZ_CP028406) highlighted a difference of 13 single-nucleotide polymorphisms (SNPs), 4 of them localized in coding regions, and only 2 indels. SNPs were validated by cross-aligning reads versus genome sequences to exclude assembling errors. This, together with the sharing of the same plasmid, has great implications for the persistence of Listeria monocytogenes in the environment and in the food industry (8). We hope that the availability of the genome sequence for these 2 strains can help in clarifying persistence mechanisms and understanding the reasons for such genome stability to prevent any reemerging event.

### Data availability.

The genome sequence of the Listeria monocytogenes serotype 1/2a 2018TE5305-4 strain was deposited at GenBank under accession number CP029372. Raw reads were submitted to the SRA repository under accession number SRR7691552.
